# Targeting PAK4 reverses cisplatin resistance in NSCLC by modulating ER stress

**DOI:** 10.1038/s41420-024-01798-7

**Published:** 2024-01-18

**Authors:** Shixin Liu, Pingshan Yang, Lu Wang, Xiaofang Zou, Dongdong Zhang, Wenyou Chen, Chuang Hu, Duqing Xiao, Hongzheng Ren, Hao Zhang, Songwang Cai

**Affiliations:** 1 Department of Thoracic Surgery, the First Affiliated Hospital of Jinan University, No.601 Huangpu Road West, Guangzhou, Guangdong 510632 China; 2 State Key Laboratory of Bioactive Molecules and Druggability Assessment, and Minister of Education Key Laboratory of Tumor Molecular Biology, Institute of Precision Cancer Medicine and Pathology, School of Medicine, Jinan University, Guangzhou, 510632 China; 3 Department of Medical Oncology, Cancer Center, Meizhou People’s Hospital (Huangtang Hospital), Meizhou Academy of Medical Sciences, Meizhou, China; 4 Guangdong Provincial Key Laboratory of Precision Medicine and Clinical Translational Research of Hakka Population, Meizhou, China; 5 Department of Pathology, Gongli Hospital, Naval Medical University, Shanghai, 200135 China; 6 Department of Pathology, Heping Hospital, Changzhi Medical College, Changzhi, 000465 China; 7 State Key Laboratory of Bioactive Molecules and Druggability Assessment, and Minister of Education Key Laboratory of Tumor Molecular Biology, Institute of Precision Cancer Medicine and Pathology, School of Medicine, Jinan University, Guangzhou; The Second Affiliated Hospital of Shantou University Medical College, Shantou, China

**Keywords:** Non-small-cell lung cancer, Cancer therapeutic resistance

## Abstract

Chemoresistance poses a significant impediment to effective treatments for non-small-cell lung cancer (NSCLC). P21-activated kinase 4 (PAK4) has been implicated in NSCLC progression by invasion and migration. However, the involvement of PAK4 in cisplatin resistance is not clear. Here, we presented a comprehensive investigation into the involvement of PAK4 in cisplatin resistance within NSCLC. Our study revealed enhanced PAK4 expression in both cisplatin-resistant NSCLC tumors and cell lines. Notably, PAK4 silencing led to a remarkable enhancement in the chemosensitivity of cisplatin-resistant NSCLC cells. Cisplatin evoked endoplasmic reticulum stress in NSCLC. Furthermore, inhibition of PAK4 demonstrated the potential to sensitize resistant tumor cells through modulating endoplasmic reticulum stress. Mechanistically, we unveiled that the suppression of the MEK1-GRP78 signaling pathway results in the sensitization of NSCLC cells to cisplatin after PAK4 knockdown. Our findings establish PAK4 as a promising therapeutic target for addressing chemoresistance in NSCLC, potentially opening new avenues for enhancing treatment efficacy and patient outcomes.

## Introduction

Globally, lung cancer is currently the major cause of cancer-related death, and most of these are due to non-small cell lung cancers (NSCLC). Currently, NSCLC can be treated most effectively by complete resection surgery. However, most people diagnosed with NSCLC have missed the opportunity for surgery by the time of diagnosis [1, 2]. Chemotherapy is the primary method to treat individuals with advanced NSCLC, and chemotherapy that uses cisplatin is the mainstay of first-line therapy [3]. Cisplatin directly kills cancer cells by inducing apoptosis. However, prolonged use of cisplatin often leads to drug resistance, which consequently results in unsuccessful treatment, as evidenced by either tumor progression or recurrence [4], ultimately leading to death events [5]. Cisplatin resistance often occurs due to a cellular defense mechanism that confers resistance by reducing the ability to mediate apoptosis, enhancing the repair of DNA damage, altering cell cycle checkpoints, and disrupting cytoskeleton assembly [6, 7]. Nevertheless, the exact mechanisms underlying cisplatin resistance remain largely unclear [8].

P21-activated kinases (PAKs) are a family of serine/threonine (Thr) protein kinases closely related to many oncogenic signaling pathways. Various human solid malignant tumors have shown mutational activation or overexpression of Pak isoforms. PAKs play a role in regulating gene transcription, cytoskeletal reorganization, oncogenic transformation, cell proliferation, and cell invasion [9, 10]. Reports have revealed that PAKs contribute to chemoresistance in the head and neck [11], colorectal [12], pancreatic [13–15], and prostate cancers [16]. Moreover, high PAK4 levels are associated with chemoresistance in gastric cancer [17], ovarian cancer [18], and pancreatic ductal adenocarcinoma [19]. According to prior research, higher levels of P21-activated kinase 4 (PAK4) are linked to unfavorable prognosis in NSCLC and promote migration and invasion [20]. Nonetheless, the mechanism of how high PAK4 expression affects chemoresistance in NSCLC cells needs to be studied in further detail.

The endoplasmic reticulum (ER) is a perinuclear organelle that synthesizes and folds proteins. The ER stress response is stimulated by several types of cellular stress, including chemotherapy [21]. When the load exceeds the folding capacity of the ER, the cells activate the unfolded protein response (UPR) as a defense mechanism. The UPR activates signaling pathways like the PERK, IRE1/X-box binding protein-1 (XBP-1), and transcription factor-6 (ATF6). Under normal physiological conditions, UPR-induced apoptosis may eliminate a small number of cells in an ER-stressed environment that stays uncorrected in spite of the involvement of UPR [22].

The 78-kDa glucose-regulated protein (GRP78), or the immunoglobulin heavy chain binding protein (BiP), plays a vital role in maintaining ER homeostasis, along with regulating the activation of ER stress sensors and initiating the ER stress response [23]. Tumors frequently exhibit GRP78 overexpression, which protects cancer cells against ER stress and a variety of cancer therapeutic agents. GRP78 overexpression has been demonstrated to confer resistance to apoptosis mediated by chemotherapy [24]. However, the contribution of GRP78 to chemoresistance in NSCLC is yet to be determined.

The current report highlighted that high PAK4 levels are linked to cisplatin resistance in NSCLC. PAK4 knockdown sensitizes cisplatin-resistant NSCLC cells. Moreover, the findings showed that ER stress is modulated by the suppression of glucose-regulated protein 78 (GRP78). The mitogen-activated protein kinase kinase 1 (MAPKK1, MEK1)/extracellular signal-regulated protein kinase 1/2 (ERK1/2) signaling pathway mediates this process by suppressing GRP78 transcription and reducing GRP78 expression. The findings of this research provide a detailed understanding of the mechanisms involved in resistance to cisplatin in NSCLC and identify a potentially useful therapeutic target to sensitize tumors.

## Results

### Association between PAK4 levels and NSCLC cisplatin resistance

**Fig. 1:**
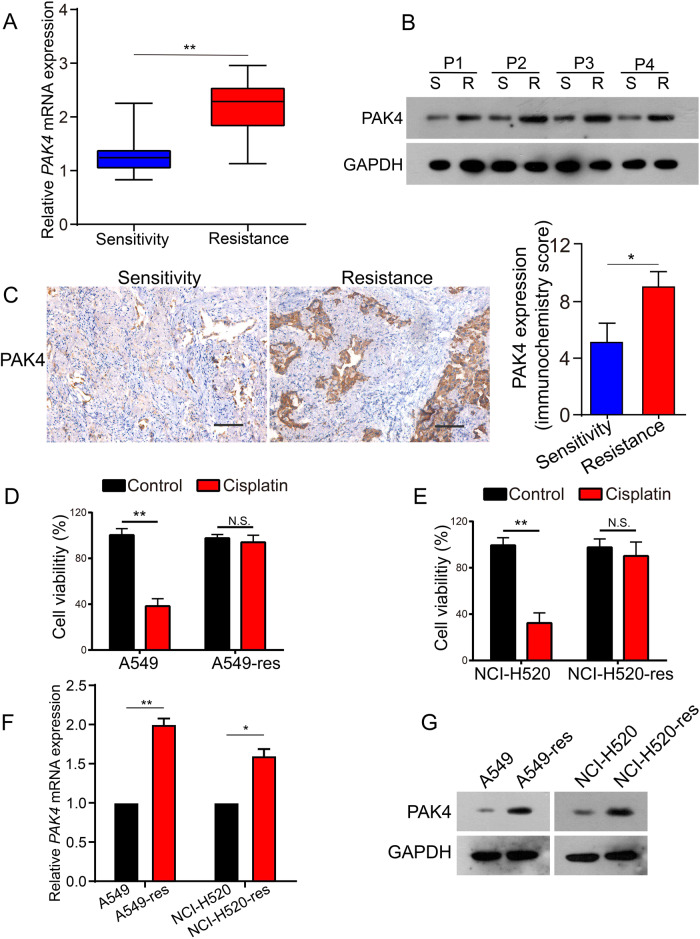
PAK4 was upregulated in cisplatin-resistant NSCLC. **A** mRNA expression of PAK4 was analyzed in 20 cisplatin-sensitive and matched cisplatin-resistant tissues. **B** Western blot analysis of PAK4 expression in the paired tissues. **C** Representative immunohistochemical staining (left panels) and statistical analysis (right panels) were shown in 55 cisplatin-sensitive NSCLC tissues and 104 cisplatin-resistant NSCLC tissues. Scale bars 100 μm. **D**, **E** Cells were treated with 5 μmol/L cisplatin for 48 h and cell viability was measured by CCK8 assay. **F**, **G** Real-time PCR and western blot were analyzed PAK4 levels. Data are shown as the means of three independent experiments or representative data. Data are expressed as mean ± standard deviation. *P < 0.05, **P < 0.01 by Student’s *t* test.

Previously, it was reported that PAK4 levels in NSCLC were considerably elevated compared to healthy lung tissue and associated with poor prognosis [20]. Western blot and qPCR were conducted to identify the correlation between the expression of PAK4 and chemoresistance in NSCLC, using 20 primary cisplatin-sensitive and 20 matched cisplatin-resistant NSCLC tissues as samples (after chemotherapy, the patients became cisplatin-resistant). PAK4 had elevated levels of mRNA and protein in NSCLC tissues that were resistant to cisplatin (Fig. 1A, B). To further confirm whether PAK4 levels were elevated in cisplatin-resistant NSCLC tissues, 159 human NSCLC tissues (55 tissues sensitive to cisplatin and 104 tissues resistant to it) were stained immunohistochemically. According to the results, protein levels of PAK4 in NSCLC tissues that showed cisplatin resistance were significantly elevated (Fig. 1C, *P* < 0.05).

To observe the link between PAK4 expression and chemoresistance in NSCLC in detail, the mRNA and protein levels of PAK4 were assessed in cisplatin-sensitive NSCLC cells (A549 and NCI-H520 cells) and cisplatin-resistant NSCLC cells (A549-res and NCI-H520-res cells) using qPCR and western blotting. A549-res and NCI-H520-res cells were established as explained earlier [25, 26]. After treatment with 5 μmol/L cisplatin in vitro, cell viability was observed using the CCK8 assay. Considerable resistance to the pharmacological cytotoxicity of cisplatin was observed in A549-res and NCI-H520-res cells in contrast with their parental cells (Fig. 1D, E). Furthermore, elevated mRNA and protein levels of PAK4 were observed in NSCLC cells with cisplatin- resistance in contrast with their parental cells (Fig. 1F, G). These outcomes collectively indicate that elevated PAK4 levels might be linked to cisplatin resistance in NSCLC.

### Enhanced chemosensitivity in cisplatin-resistant NSCLC cells due to PAK4 knockdown

**Fig. 2:**
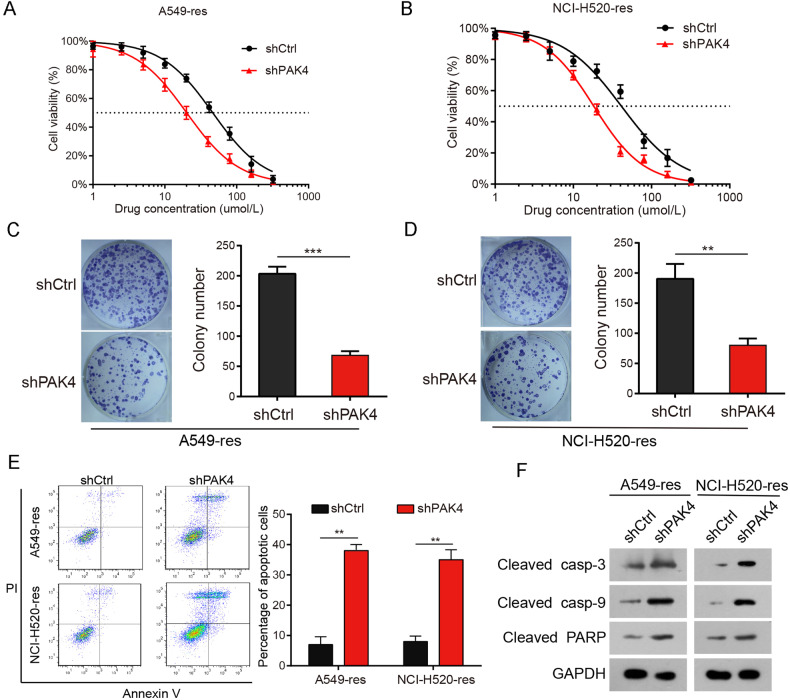
PAK4 knockdown enhanced NSCLC cell chemosensitivity. **A**, **B** A549-res cells (**A**) and NCI-H520-res cells (**B**) stably expressing PAK4 shRNA (shPAK4) or control shRNA (shCtrl) were treated with the indicated doses of cisplatin for 48 h and cell viability was measured by CCK8 assay. Data are expressed as mean ± SEM. **C**, **D** A549-res cells and NCI-H520-res cells stably expressing PAK4 shRNA (shPAK4) or control shRNA (shCtrl) were treated with cisplatin (5 μmol/L) for 48 h. Colony-forming ability for A549-res cells (**C**). Colony-forming ability for NCI-H520-res cells (**D**). **E** Flow cytometry analyses of the percentages of apoptotic cells. **F** The levels of apoptosis markers (cleaved casp-3, cleaved casp-9 and cleaved PARP) were examined by western blot analysis. Data are shown as the means of three independent experiments or representative data. Data are expressed as mean ± standard deviation. **P < 0.01, ***P < 0.001 by Student’s *t* test.

Based on these results, it is noteworthy that PAK4 might be significantly involved in cisplatin resistance in NSCLC. To confirm this, stable PAK4 knockdown (shPAK4) cells were constructed using A549-res and NCI-H520-res cells (Supplementary Fig. 1A). The cells were incubated with varied concentrations of cisplatin (2.5, 5, 10, 20, 40, 60, 80, and 100 μmol/L). The IC50 values for cisplatin in A549-res and NCI-H520-res cells stably expressing shPAK4 were reduced when compared with those in shCtrl cells (Fig. 2A, B). Colony formation assays showed that PAK4 knockdown remarkably inhibited the colony-forming abilities of A549-res and NCI-H520-res cells (Fig. 2C, D). Furthermore, after treatment with 5 μmol/L cisplatin in vitro, PAK4 depletion significantly enhanced the proportion of apoptotic cells (Fig. 2E). Moreover, the proportion of apoptosis markers (cleaved caspase-3, cleaved caspase-9, and cleaved PARP) was remarkably enhanced in PAK4-depleted cells (Fig. 2F). These data show that PAK4 knockdown enhanced apoptosis in cisplatin-resistant NSCLC cells. Similar outcomes were noted in cisplatin-sensitive NSCLC cells (A549 and NCI-H520) (Supplementary Fig. 1B–E). These results from laboratory experiments suggest that PAK4 knockdown promoted the sensitivity of NSCLC cells to cisplatin.

### Cisplatin evoked ER stress-inducing apoptosis in NSCLC cells

**Fig. 3:**
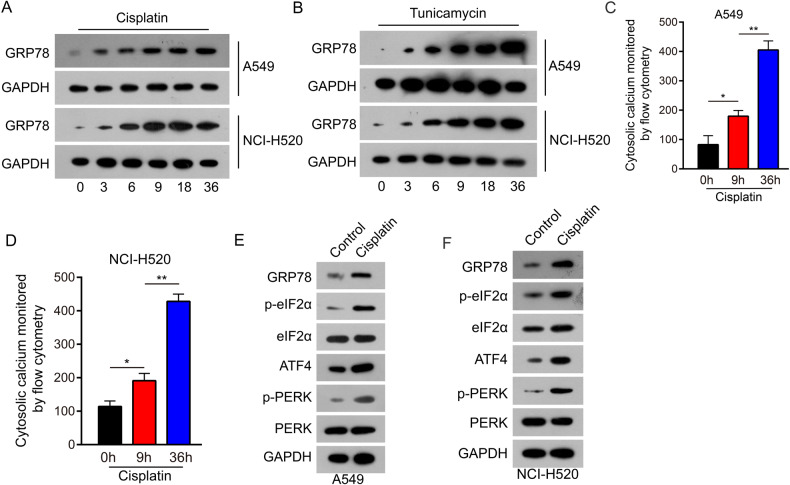
Cisplatin evoked ER stress in NSCLC cells. **A** GRP78 expression was examined in A549 and NCI-H520 cells following treatment with cisplatin (5 μmol/L). Floating and adherent cells were lysed at 0, 3, 6, 9, 18, and 36 h and analyzed by western blot analysis. **B** GRP78 expression was examined in A549 and NCI-H520 cells following treatment with tunicamycin (200 ng/μl). Floating and adherent cells were lysed at 0, 3, 6, 9, 18, and 36 h and analyzed by western blot analysis. **C**, **D** Treated with 5 µmol/L cisplatin for 0, 9, and 36 h, A549 cells (**C**) and NCI-H520 cells (**D**) were loaded with Fluo-3am for 30 min. Cytoplasmic calcium level was examined by flow cytometry assay. Data are expressed as mean ± standard deviation. **E**, **F** Molecular markers of the endoplasmic reticulum stress signaling pathway were detected by western blot analysis in A549 and NCI-H520. Data are shown as the means of three independent experiments or representative data. Data are expressed as mean ± standard deviation. *P < 0.05, **P < 0.01 by Student’s *t* test.

Cisplatin is a widely used and effective cytotoxic drug used for treating solid tumors. It reacts with nucleophilic sites on intracellular macromolecules to form DNA, RNA, and protein adducts and is believed to kill cells by binding to DNA and interfering with its repair, thus activating DNA-damage checkpoints [27]. Chemotherapeutic drugs have been shown to induce apoptosis via ER stress [28, 29]. Hence, we investigated the ability of cisplatin to induce apoptosis independently of DNA damage. Therefore, we examined whether cisplatin could induce ER stress in NSCLC cells. A549 and NCI-H520 cells were exposed to 5 μmol/L cisplatin, and the levels of the classic ER stress inducer GRP78 enhanced in a time-dependent manner (Fig. 3A). Similar alterations were detected in parallel time-course experiments after administering tunicamycin (200 ng/μl), a well-known initiator of ER stress and apoptosis (Fig. 3B).

The ER is an intracellular organelle that performs multiple functions, including the maintenance of calcium homeostasis. Calcium disorders can result in ER stress [30]. This study assessed cytoplasmic calcium levels using flow cytometry. Time-dependent elevated cytoplasmic calcium levels were detected in A549 (Fig. 3C, Supplementary Fig. 3A) and NCI-H520 cells (Fig. 3D, Supplementary Fig. 3B). Furthermore, the downstream signaling pathway of GRP78 was examined after treatment with cisplatin (5 μmol/L) for 36 h. The results indicated that the levels of phosphorylation of PERK (p-PERK), eIF2α (p-eIF2α), and ATF4 increased consistently with the level of GRP78 (Fig. 3E, F). These data show that cisplatin stimulated ER stress in NSCLC cells.

### PAK4 knockdown chemosensitized cisplatin-resistant NSCLC cells through downregulation of GRP78

Cisplatin induces apoptosis in NSCLC cells through ER stress-mediated by GRP78 upregulation. However, during chronic ER stress, GRP78 overexpression may also contribute to adaptive mechanisms such as translational attenuation and autophagy to promote cellular survival and chemoresistance [31].

**Fig. 4:**
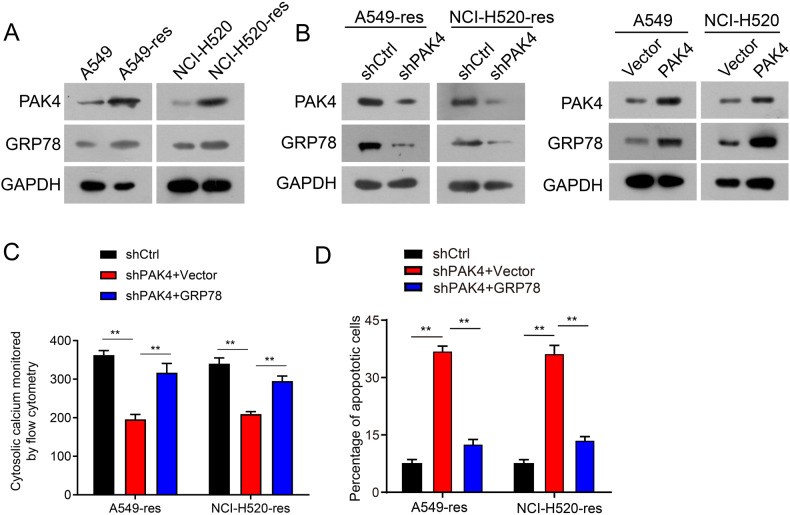
PAK4 knockdown chemosensitized NSCLC cells via modulating ER stress. **A** PAK4 and GRP78 expression profiles were examined by western blot analysis in A549/A549-res and NCI-H520/NCI-H520-res cells. **B** A549-res and NCI-H520-res cells stably expressing PAK4 shRNA (shPAK4) or control shRNA (shCtrl) were treated with 5 µmol/L cisplatin for 48 h. PAK4 and GRP78 expression was examined by western blot analysis (left panel) and Western blot analyses of PAK4 and GRP78 expression in PAK4 overexpressed cells (right panel). **C** A549-res and NCI-H520-res cells stably expressing PAK4 shRNA (shPAK4) or control shRNA (shCtrl) or shPAK4 and GRP78 plasmids were treated with 5 µmol/L cisplatin for 48 h. Cytoplasmic calcium level was examined by flow cytometry assay. **D** A549-res and NCI-H520-res cells stably expressing PAK4 shRNA (shPAK4) or control shRNA (shCtrl) were transfected with GRP78 or control vector, followed by treatment with 5 µmol/L cisplatin for 48 h. Apoptosis rates were determined by flow cytometry. Data are shown as the means of three independent experiments or representative data. Data are expressed as mean ± standard deviation. **P < 0.01, ***P < 0.001 by Student’s *t* test.

It was hypothesized that the downregulation of GRP78 through PAK4 depletion would make cisplatin-resistant NSCLC cells more sensitive to cisplatin. First, ER stress levels were examined in cisplatin-resistant NSCLC cells (A549-res and NCI-H520-res cells) and parental cell cells (A549 and NCI-H520 cells) by western blotting. The results showed that the levels of GRP78 were elevated in cisplatin-resistant NSCLC cells, consistent with the levels of PAK4 (Fig. 4A). To further explore whether ER stress is involved in cisplatin resistance associated with PAK4 overexpression in NSCLC, GRP78 expression was assessed using western blotting and qPCR. The outcomes revealed that PAK4 knockdown decreased GRP78 expression in A549-res and NCI-H520-res cells (Fig. 4B and Supplementary Fig. 2A), whereas overexpression of PAK4 significantly increased GRP78 mRNA and protein levels in both A549 and NCI-H520 cells (Fig. 4B and Supplementary Fig. 2B). Cytoplasmic calcium levels and apoptosis rates were significantly decreased in A549-res and NCI-H520-res cells transfected with shPAK4 (Fig. 4C, D and Supplementary Fig. 3C, D). Moreover, the effects of PAK4 knockdown on cytoplasmic calcium levels and apoptosis in A549-res and NCI-H520-res cells were attenuated by GRP78 overexpression (Fig. 4C, D and Supplementary Fig. 3C, D). These findings highlight that PAK4 knockdown improved drug sensitivity in cisplatin-resistant NSCLC cells by downregulating GRP78.

### PAK4 knockdown chemosensitized cisplatin-resistant NSCLC xenografts in vivo

**Fig. 5:**
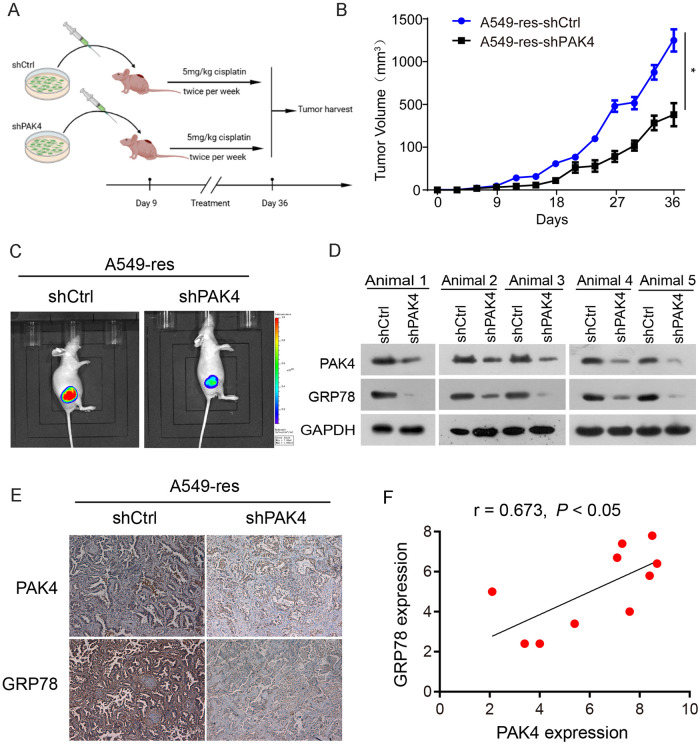
PAK4 knockdown chemosensitized cisplatin-resistant NSCLC xenografts in vivo. **A** Scheme indicating the timing of xenografting and longitudinal treatment. **B** A549-res cells stably expressing shPAK4 and luciferase reporter were injected subcutaneously into nude mice. Mice were intraperitoneally injected with cisplatin (5 mg/kg) twice per week once the tumors were established. Tumor growth was monitored with a standard caliper. **C** In vivo bioluminescence imaging was shown on day 36. **D** PAK4 and GRP78 expression profiles were examined by western blot in xenograft tumors. **E** Representative IHC staining results of PAK4 and GRP78 were examined in xenograft tumors. **F** A positive correlation between PAK4 and GRP78 expression was observed by IHC staining in xenograft tumors. Data are expressed as mean ± standard deviation.

To assess whether PAK4 knockdown could chemosensitize NSCLC cells in vivo, A549-res cells stably expressing shPAK4 and shCtrl were administered subcutaneously into the right lateral hips of nude mice (Fig. 5A). The size of the tumor was measured using standard calipers and bioluminescence imaging. Cisplatin was administered to the mice via intraperitoneal injection twice per week. As shown in Fig. 5B, C, the growth of cancers generated by cisplatin-resistant cells (A549-res cells) was significantly suppressed in the PAK4 knockdown group. Moreover, bioluminescence imaging also showed that PAK4 knockdown significantly suppressed tumor growth and improved chemosensitivity to cisplatin (Fig. 5B, C).

Furthermore, GRP78 expression in orthotopic tumors generated by A549-res cells was significantly downregulated in the PAK4 knockdown group, as indicated by both western blot analysis (Fig. 5D) and immunohistochemistry (Fig. 5E). GRP78 expression positively correlated with PAK4 expression in orthotopic tumors generated by A549-res cells (Fig. 5F). These findings indicated that GRP78 is critically involved in PAK4-induced cisplatin-resistant NSCLC.

### GRP78 expression was positively linked to PAK4 expression in NSCLC tumor tissues

**Fig. 6:**
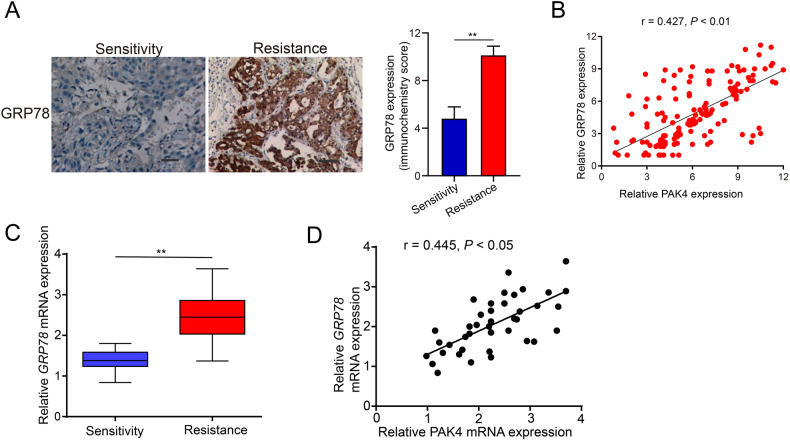
PAK4 and GRP78 expression profiles in NSCLC tissues. **A** Representative immunohistochemical staining (left panels) and statistical analysis (right panels) of GRP78 protein expression difference between 55 cisplatin-sensitive NSCLC tissues and 104 cisplatin-resistant NSCLC tissues. **B** The correlation between PAK4 expression and GRP78 expression in 159 human NSCLC tissues was examined by IHC staining. **C** The relative mRNA expression of the GRP78 in 20 cisplatin-sensitive NSCLC tissues and matched cisplatin-resistant NSCLC tissues was examined by Real-time PCR. **D** The correlation between relative PAK4 mRNA expression and relative GRP78 mRNA expression in 20 cisplatin-sensitive NSCLC tissues and matched cisplatin-resistant NSCLC tissues. Data are shown as the means of three independent experiments or representative data. Data are expressed as mean ± standard deviation. **P < 0.01 by Student’s *t* test.

To further elucidate the mechanism between PAK4 and GRP78 in cisplatin resistance in NSCLC, we examined GRP78 expression using an IHC assay. The data revealed that GRP78 levels were remarkably elevated in cisplatin-resistant NSCLC tissues (Fig. 6A). The outcomes of Pearson’s correlation analysis highlighted a notable positive association between PAK4 and GRP78 (*r* = 0.427, *P* < 0.01; Fig. 6B). Furthermore, the relative GRP78 mRNA expression was examined in 20 cisplatin-sensitive NSCLC tissues and corresponding cisplatin-resistant NSCLC tissues using qPCR. According to the outcomes, GRP78 mRNA expression level was remarkably elevated in cisplatin-resistant NSCLC tissues (Fig. 6C), and Pearson’s correlation analysis revealed a remarkably positive link between GRP78 and PAK4 mRNA expression (*r* = 0.445, *P* < 0.05; Fig. 6D). Based on these outcomes. it can be deduced that GRP78 expression is closely linked to the expression of PAK4 in NSCLC.

### PAK4 knockdown downregulated GRP78 expression through suppressing MEK1 activation

The activated MEK/ERK signaling pathway is a crucial cause of resistance to apoptosis in malignant tumors, and some studies have reported that downregulating pERK expression suppresses GRP78 transcription and reduces its expression [32–34].

**Fig. 7:**
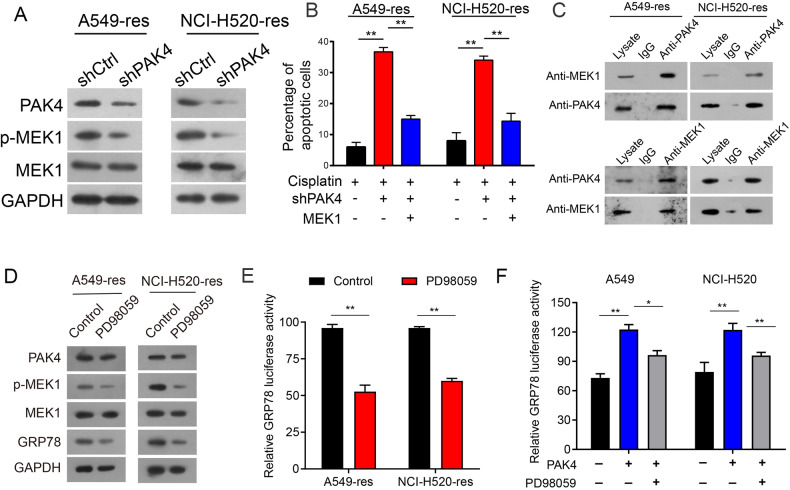
PAK4 knockdown inhibited GRP78 expression. **A** PAK4, GRP78, p-MEK1, and MEK1 expression were examined by western blot. **B** Flow cytometry analyses of the percentages of apoptotic cells in A549-res and NCI-H520-res cells transfected with the shPAK4 or MEK1 plasmids or in combinations between shPAK4 and MEK1. **C** A549-res and NCI-H520-res cell lysates were immunoprecipitated with PAK4 antibody and subjected to Western blotting to ascertain MEK1 and PAK4 expression using indicated antibodies (upper panels). Cell lysates were immunoprecipitated with MEK1 antibody (lower panels). **D** A549-res cells and NCI-H520-res cells were treated with PD 98059 or control for 48 h, and PAK4, MEK1, p-MEK1, and GRP78 expression profiles were examined by western blot. **E** A549-res cells and NCI-H520-res cells were treated with MEK1 inhibitor (PD 98059). Luciferase activity of GRP78 was examined after 48 h. **F** Luciferase activity of GRP78 was examined in the overexpression of PAK4 cells (A549 and NCI-H520) with or without PD 98059. Data are shown as the means of three independent experiments or representative data. Data are expressed as mean ± standard deviation. *P < 0.05, **P < 0.01 by Student’s *t* test.

Therefore, it is hypothesized that downregulation of PAK4 leads to a reduction in GRP78 expression through the inhibition of MEK1 activity. To validate this proposed mechanism, western blotting demonstrated that depletion of PAK4 led to a decrease in phosphorylated MEK1 levels, while total MEK1 levels remained unaffected in both A549-res and NCI-H520-res cells (Fig. 7A). Moreover, quantitative PCR (qPCR) indicated that there was no impact on the MEK1 mRNA levels (Supplementary Fig. 3E). Additionally, the introduction of MEK1 overexpression in PAK4 knockdown cells was observed to diminish the percentage of apoptotic cells (Fig. 7B and Supplementary Fig. 3F). Co-immunoprecipitation (co-IP) results further revealed a specific interaction between PAK4 and MEK1 in A549-res and NCI-H520-res cells (Fig. 7C). As expected, the MEK1 inhibitor (PD 98059) treatment specifically reduced the phosphorylation level of MEK1 while leaving the expression level of MEK1 largely unaffected (Fig. 7D). Treatment with PD 98059 was found to suppress the transcriptional activity of the GRP78 promoter in both A549-res and NCI-H520-res cells (Fig. 7E). In addition, luciferase assays indicated that PAK4 influenced the transcriptional activity of the GRP78 promoter, and this process was suppressed by PD 98059 (Fig. 7F). Collectively, these findings suggest that PAK4 knockdown impedes GRP78 expression through the suppression of the MEK1 signaling pathway in NSCLC cells.

## Discussion

PAK4 is a vital oncogene in various malignant tumors. However, it remains unclear whether high PAK4 expression contributes to chemoresistance in NSCLC. In this study, it was highlighted that PAK4 levels are elevated in cisplatin-resistant NSCLC tissues and cells. Furthermore, PAK4 knockdown significantly sensitized cisplatin-resistant NSCLC cells both in laboratory experiments as well as in vivo. These outcomes reveal that PAK4 is crucially involved in cisplatin resistance in NSCLC.

Recent studies have highlighted that PAK4 regulates multiple pathways to sustain tumor cell malignancy and confers drug resistance. In pancreatic cancer, PAK4 maintains stem cell-like phenotypes and decreases chemosensitivity to gemcitabine toxicity by activating STAT3 signaling [35]. The PI3K/AKT pathway is activated by PAK4 and facilitates cisplatin resistance in cells of cervical cancer [36]. PAK4 confers cisplatin resistance in gastric cancer cells via PI3K/AKT and MEK/ERK-dependent pathways [37]. However, the molecular mechanism of PAK4-mediated chemoresistance in NSCLC remains unclear.

Intrinsic or acquired resistance to cisplatin in cells is mediated by multiple mechanisms such as decreasing the accumulation of cisplatin in cells, increasing drug metabolism and inactivation by detoxification enzymes, increasing DNA repair capacity, and inactivating apoptosis programs. Cisplatin induces DNA lesions and repair mechanisms that involve multiple proteins and pathways, including cell stresses [38–40]. Extrinsic cellular stresses, such as nutrient deprivation, glucose starvation, low pH, and hypoxia, resulting in the accumulation of unfolded or aberrantly folded proteins in the ER lumen. The accumulation of misfolded proteins triggers ER stress, which induces a signaling cascade known as the unfolded protein response (UPR) [41]. Cisplatin therapy has been reported to stimulate ER stress in cancer cells, which is in accordant with the outcomes of this study [42, 43].

GRP78, an ER chaperone protein that serves as the master regulator of the UPR, is an intracellular protein found in the ER lumen, and cisplatin therapy can induce ER stress-mediated by GRP78 upregulation. However, in the context of a tumor environment that is characterized by abnormal stress, GRP78 expression is significantly upregulated on the surface of tumor cells, and this protein may facilitate cancer growth and contribute to drug resistance. GRP78 has been found to be upregulated and linked to drug resistance and malignancy in the brain [44–46], liver [47, 48], lung [49, 50], and breast cancer [51, 52] and in pancreatic ductal adenocarcinoma [53].

GRP78 overexpression in drug resistance mainly involves a reduction in drug-induced apoptosis [28]. Previous research showed that GRP78 overexpression contributed to cisplatin resistance in lung cancer, but the relevant underlying is yet to be studied in further detail [54, 55]. This study showed that high GRP78 expression suppressed cisplatin sensitivity in NSCLC cells. In addition, it was found that GRP78 was involved in cisplatin resistance and was associated with high PAK4 expression. Based on the available literature, there are no other reports on the involvement of ER stress in high PAK4 expression-induced cisplatin resistance in malignant tumors. It was discovered that high PAK4 expression enhanced GRP78 transcription by activating the MEK1/ERK1/2 signaling pathway, thereby inducing cisplatin resistance in NSCLC. These findings may directly affect the clinical management of individuals with PAK4-overexpressing NSCLC and provide a new strategy for targeting pivotal ER chaperone proteins for cancer therapy. However, the present study has certain limitations. Firstly, it should be noted that this study has examined multiple cells and mouse models including PAK4 overexpression studies. It should be noted that these results cannot be used to determine the efficiency of targeting PAK4 in patients with PAK4-overexpressing cancer. Large independent prospective clinical studies are needed to validate these results. Follow-up studies will be conducted to further investigate these issues. These findings offer valuable insights into the clinical application of the mechanisms via which different oncogenes regulate chemotherapy resistance.

## Conclusions

The findings of this study demonstrated that PAK4 knockdown promoted cisplatin sensitivity in NSCLC by modulating ER stress through the suppression of GRP78 expression. Therefore, PAK4 might be a valuable therapeutic target for NSCLC chemoresistance.

## Materials and methods

### Tissue specimens and cell culture

Cisplatin-sensitive and -resistant tissues were retrieved from individuals with NSCLC. Cisplatin-sensitive NSCLC tissue samples from 20 patients and 20 matched cisplatin-resistant NSCLC tissues from the same patients (after chemotherapy, patients became cisplatin-resistant) were collected between January 2016 and May 2021 at the First Affiliated Hospital of Jinan University. Isolated tissues were stored in liquid nitrogen. A total of 159 NSCLC samples, including 55 cisplatin-sensitive and 104 cisplatin-resistant NSCLC tissues, were retrieved from January 2008 to January 2021. Histopathological tests were performed in order to ensure definite diagnoses. The patient received first-line chemotherapy (platinum-based agents). The tumor was comprehensively evaluated by imaging-related examination after two cycles of therapy (6 weeks). The Response Evaluation Criteria in Solid Tumors (RECIST 1.1) was utilized to observe the objective response of the tumor [56]. Complete response (CR) and partial response (PR) were enrolled in the cisplatin-sensitive patient group, and progressive disease (PD) was enrolled in the cisplatin-resistant patient group. Approval for this research was granted by the Institutional Research Ethics Committee of the First Affiliated Hospital of Jinan University. Human NSCLC cell lines A549 and NCI-H520 were retrieved from the Cell Bank of the Chinese Academy of Sciences. STR was utilized to identify these cells, which were confirmed to be free of mycoplasma and grown in RPMI 1640 containing 10% newborn calf serum (Gibco, Invitrogen Life Technologies, USA).

### Construction of cisplatin-resistant NSCLC cell lines

The A549 and NCI-H520 NSCLC cells were supplied by the Cell Bank of the Chinese Academy of Sciences. In order to develop cisplatin-resistant A549 and NCI-H520 cells, they were incubated with cisplatin as explained before [25, 26]. Briefly, gradient cisplatin concentrations were used to treat parental cells (Sigma-Aldrich). To maintain the cisplatin-resistant phenotype of A549 and NCI-H520 cells, 5μmol/L cisplatin was added to 1640 medium. The cells were named A549-res and NCI-H520-res, respectively.

### RNA extraction and quantitative real-time PCR

For RNA extraction, TRIzol reagent (Invitrogen Life Technologies, USA) was utilized. cDNA synthesis was carried out with the aid of the PrimeScript RT Reagent Kit (Promega, USA) [57–59]. ABI 7900HT Fast Real-Time PCR system (Applied Biosystems) was utilized to perform qPCR. The primer sequences utilized are stated below: PAK4 forward (5′-ATGTGGTGGAGATGTACAACAGCTA-3′) and reverse (5′-GTTCATCCTGGTGTGGGTGAC-3′); GRP78 forward (5′-GGTGAAGAGGATACAG-CAGAAA-3′) and reverse (5′-GACACAGCTAGGGTTCACAATA-3′); and U6 forward (5′-TGCGGGTGCTCGCTTCGGCAGC-3′) and reverse (5′-CCAGTGCAGGGTCCGAGGT-3′).

### Vector and shRNAs construction

GRP78 and MEK1 plasmids were provided by GenePharma (China). Moreover, Santa Cruz Biotechnology (USA) provided the GRP78 and Vector, as well as produced and validated lentivirus particles expressing PAK4 shRNA or control shRNA.

### Western blot

The primary antibodies used were as follows: PAK4 (1:1000; Cell Signaling Technology), GRP78 (1:500; Cell Signaling Technology), cleaved caspase-9 (1:1000; Cell Signaling Technology), cleaved caspase-3 (1:1000; Cell Signaling Technology), cleaved PARP (1:500; Santa Cruz Biotechnology), MEK1 (1:500; Abcam), extracellular signal-regulated protein kinase 1/2 (ERK1/2) (1:500, Cell Signaling Technology), and GAPDH (1:8000, Cell Signaling Technology). Three independent experiments were performed for all western blotting studies.

### Immunohistochemistry assay

The standard streptavidin-biotin-peroxidase complex method (EnVision™ Detection System; Denmark) was used to perform immunohistochemical staining [60–63]. NSCLC tissues were cut into 4-mm sized sections, xylene was utilized to deparaffinize them, and graded ethanol series was used for rehydration. To stain them, they were treated with anti-PAK4 (1:500; Cell Signaling Technology) or GRP78 (1:500; Abcam) and subjected to incubation at 4 °C overnight. Two skilled observers independently graded the degree of immunostaining. The intensity and frequency of staining were estimated as previously described [20]. The staining index scores ranged from 0 to 12.

### Cell viability assay

In total, cells (2 × 10^3^ cells/well) were seeded into individual wells with 96-well plates. After 24 h, they were exposed to cisplatin (5 μmol/L concentration) [64–67]. After 48 h of treatment, the cell viability was quantified by means of CCK-8 assay (MedChemExpress). Stably transfected cells were seeded in 96-well plates (5,000 cells per well). After allowing the cells to attach, they were treated with various concentrations of cisplatin ranging from 0, 2.5, 5, 10, 20, 40, 60, 80, to 100 μmol/L for 48 h. Following the cisplatin treatment, 10 μL CCK-8 solution was added to each well, and the plates were incubated at 37 °C in the dark. The cell viabilities were measured at 450 nm using a Microplate Reader (BioTek). The IC50 values were determined using the SPSS 17.0 software.

### Colony formation assay

In total, cells (5 × 10^2^ cells/well) were seeded into 6-well plates. After a period of 14 days, 0.1% crystal violet was utilized for colony fixation and staining.

### Intracellular Ca^2+^ detection

After cisplatin treatment, A549 and NCI-H520 cells were treated with Fluo-3am (Beyotime Shang Hai, China) for 30 min. Flow cytometry was utilized to examine the fluorescence intensity of Fluo-3 in combination with Ca^2+^.

### Apoptosis assay

Cisplatin (5 μmol/L) was administered into the culture medium for a period of 48 h, after which the cells were collected for further analysis. Their rate of apoptosis was evaluated utilizing the Annexin V-FITC/PI Apoptosis Detection Kit (KeyGEN, China).

### Co-immunoprecipitation

To solubilize A549-res and NCI-H520-res cells (6 × 10^6^), they were immersed into 400 μl of cell lysis buffer at 4 °C for 10 min. The cell lysates were subjected to brief sonication and centrifugation, and the resulting extract was subjected to immunoprecipitation using 4 μg of either PAK4 (Cell Signaling Technology) or MEK1 (Abcam) antibodies. The sample was incubated with 60 μl of protein G plus/protein A-agarose at 4 °C with continuous inversion for 16 h. The immunocomplexes were pelleted, followed by a washing step that was repeated thrice. After incubation, the precipitated immunocomplexes were boiled in Laemmli buffer and then analyzed using western blotting with either an anti-MEK1 or anti-PAK4 antibody.

### Luciferase reporter assay

A pGL3 luciferase plasmid was developed, which contained GRP78-responsive elements upstream of the firefly luciferase reporter gene. The cells were then cotransfected with the pGL3 luciferase plasmid and pRL-TK (Promega, Madison, WI, USA) in a 48-well plate. After 48 h, the cells were harvested, and the Dual Luciferase Assay System (Promega) was used to measure luciferase activity.

### Mouse xenograft study

A549-res cells stably expressing either an empty vector or PAK4 shRNA and a luciferase reporter were generated by retroviral transduction. These cells were then injected subcutaneously into the right lateral hips of male athymic nude mice, aged 6 weeks. Once the tumors were established (approximately 40 mm^3^), the mice were given cisplatin (5 mg/kg) intraperitoneally twice a week. Bioluminescence imaging was conducted on mice every 6 days using a charge-coupled device camera (IVIS; Xenogen Corp), and the data were assessed with the IVIS Living Image software (Xenogen Corp). After 39 d of implantation, the mice were humanely euthanized by cervical dislocation. The sizes of the xenograft tumor were measured every 3 days after cisplatin treatment using a common caliper. The formula (volume = length × width^2^ × 0.52) was used to assess tumor volume. Approval for all experiments on animal models was granted by the Medicine Institutional Animal Care and Use Committee of the First Affiliated Hospital of Ji Nan University.

### Statistical analysis

SPSS 17.0 was utilized to assess all study data. All data results were confirmed from at least three independent experiments. The mean ± SD was used to present the results of the cell line experiments, and a two-tailed Student’s t-test was carried out to compare them. Fisher’s exact test or χ2 test was employed to compare the variables. The value of *P* < 0.05 was taken as a statistically significant value.

### Supplementary information

Supplementary Information

Original Images for Western blot

## Data Availability

All data generated or analyzed during this study are included in this published article and its additional information files.
