# Supplementary material for: Targeting PAK4 reverses cisplatin resistance in NSCLC by modulating ER stress
Source: Cell Death Discov. 2024 Jan 18;10:36. doi: 10.1038/s41420-024-01798-7 (PMC10796919; doi:10.1038/s41420-024-01798-7)
Supplement: Supplementary file 1 — Supplementary Information [file 41420_2024_1798_MOESM1_ESM.docx]

**Supplementary Information**

**Supplementary Figures and Figure legends**


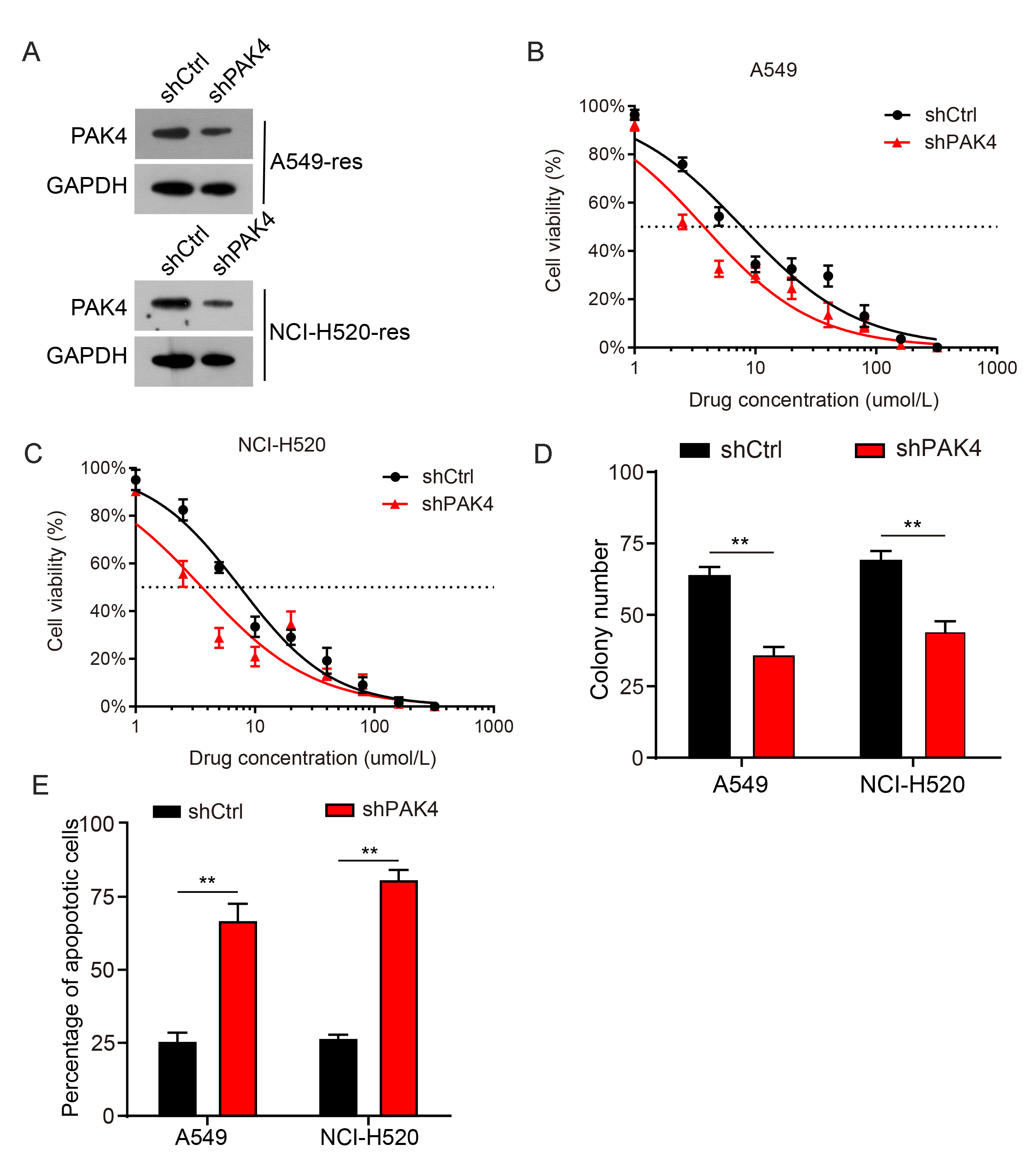


**Supplementary Figure 1.** **PAK4 knockdown enhanced cisplatin- sensitive NSCLC cell chemosensitivity. (A)** PAK4 was examined in A549 and NCI-H520 cells stably expressing shPAK4 or shCtrl by western blot analysis. **(B, C)** A549 cells (**B**) and NCI-H520 cells (**C**) stably expressing shPAK4 or shCtrl were treated with cisplatin. The indicated dose of cisplatin was added and cell viability was measured by CCK8 assay 48 h after cisplatin treatment. Data are expressed as mean ± SEM. (**D, E)** A549 cells and NCI-H520 cells stably expressing shPAK4 or shCtrl were treated with cisplatin (5 μmol/L) for 48 h. Colony-forming ability for cells **(D)**. Flow cytometry analyses of the percentages of apoptotic cells **(E)**.


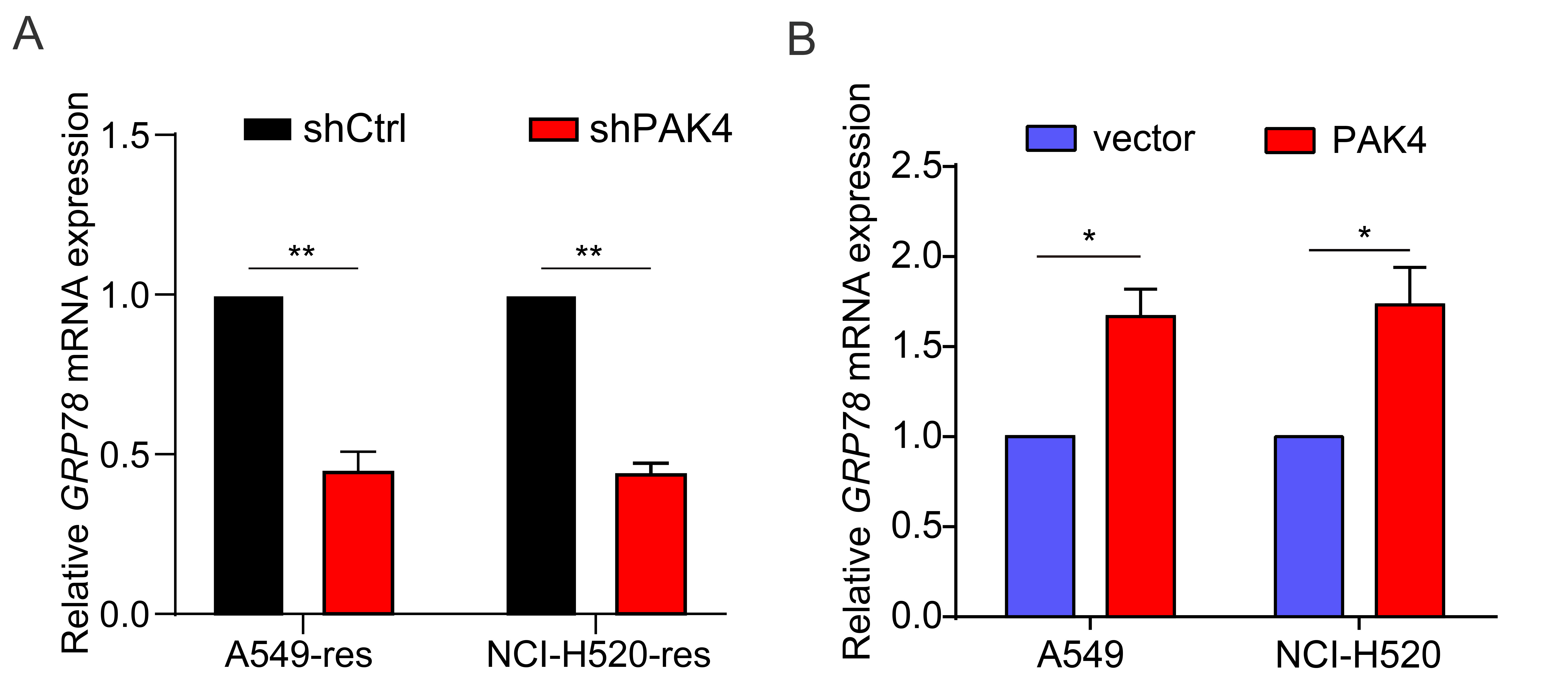


**Supplementary Figure 2.** **Cisplatin** **Activated ER stress signalling pathway in NSCLC cells.** RT-qPCR analysis of expression of GRP78 in PAK4 knockdowned cells (**A**) and PAK4 overexpressed cells (**B**). GAPDH was used as an internal control.


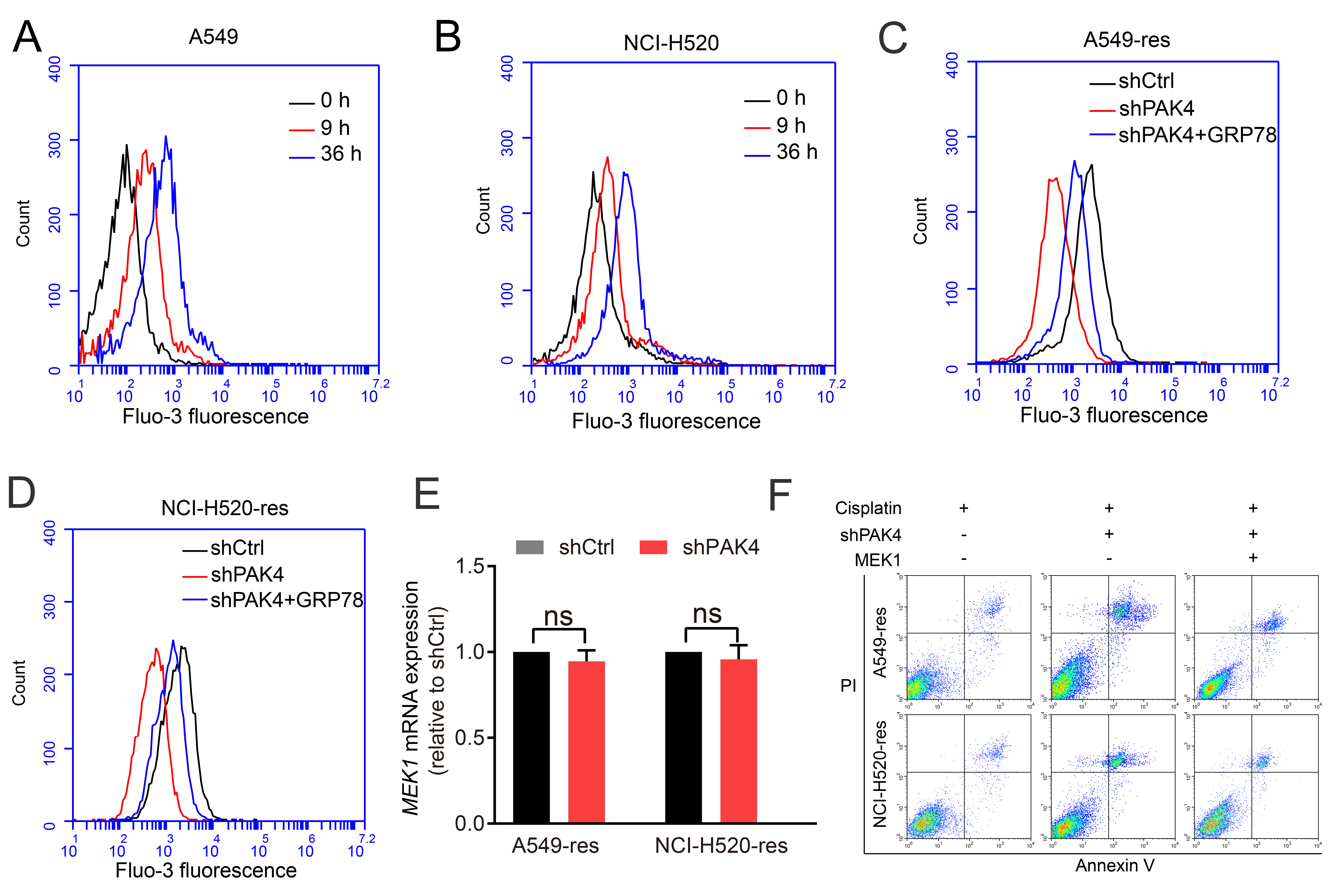


**Supplementary Figure 3.** **cytoplasmic calcium level be examined by flow cytometry assay.** Treated with 5 µmol/L cisplatin for 0, 9, 36 h, A549 cells (**A**) and NCI-H520 cells (**B**) were loaded with Fluo-3am for 30 min. Cytoplasmic calcium level was examined by flow cytometry assay. (**C**) A549-res and (**D**) NCI-H520-res cells stably expressing PAK4 shRNA (shPAK4) or control shRNA (shCtrl) were treated with 5 µmol/L cisplatin for 48 h. Cytoplasmic calcium level was examined by flow cytometry assay. (**E**) The relative mRNA expression of MEK1 in PAK4 knockout cisplatin-resistant cells was detected by Real-time PCR. (**F**) Flow cytometry analyses of the percentages of apoptotic cells in A549-res and NCI-H520-res cells transfected with the shPAK4 or MEK1 plasmids or in combinations between shPAK4 and MEK1.
