# Supplementary material for: Targeting PAK4 reverses cisplatin resistance in NSCLC by modulating ER stress
Source: Cell Death Discov. 2024 Jan 18;10:36. doi: 10.1038/s41420-024-01798-7 (PMC10796919; doi:10.1038/s41420-024-01798-7)
Supplement: Supplementary file 2 — Original Images for Western blot [file 41420_2024_1798_MOESM2_ESM.docx]

Figure1A


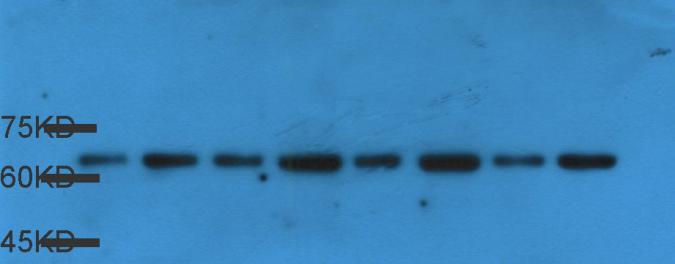


PAK4


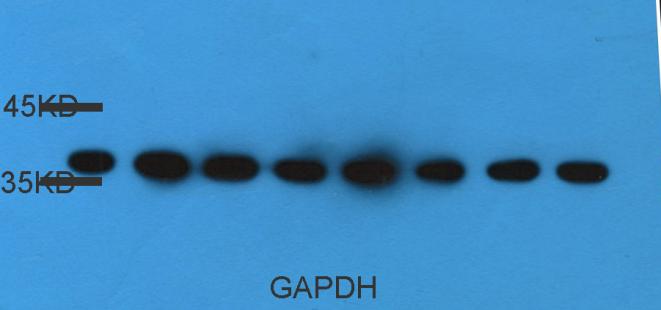


GAPDH

Figure1F


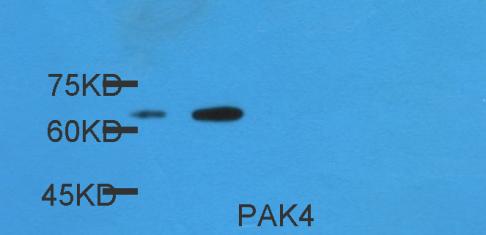

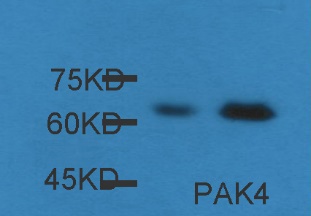


A549/A549-res NCI-H520/NCI-H520-res


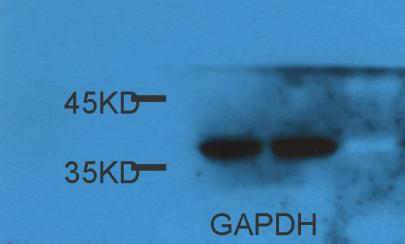

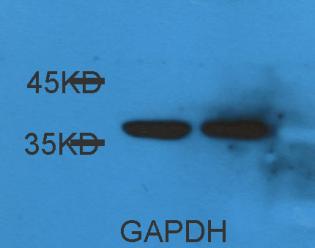


A549/A549-res NCI-H520/NCI-H520-res

Figure2F


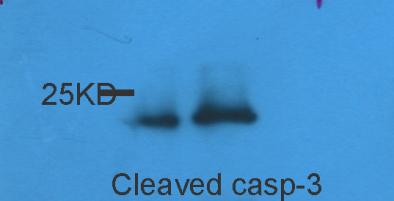

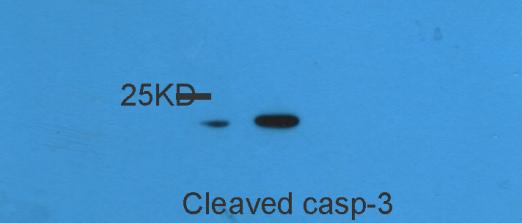


A549-res NCI-H520-res


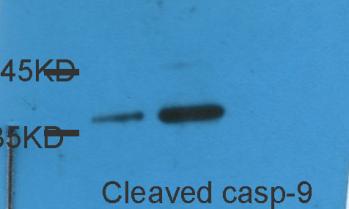

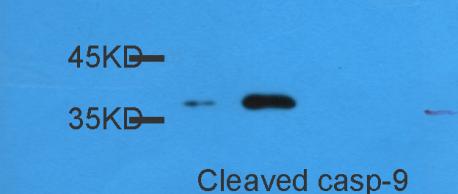


A549-res NCI-H520-res


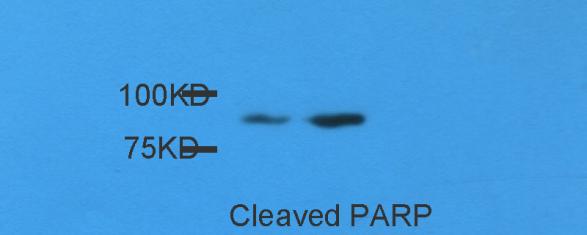

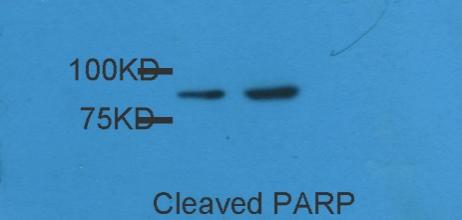


A549-res NCI-H520-res


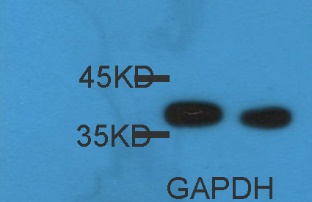

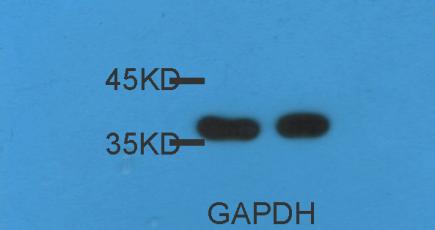


A549-res NCI-H520-res

Figure3A


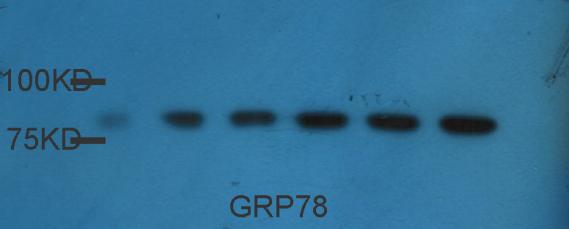


A549


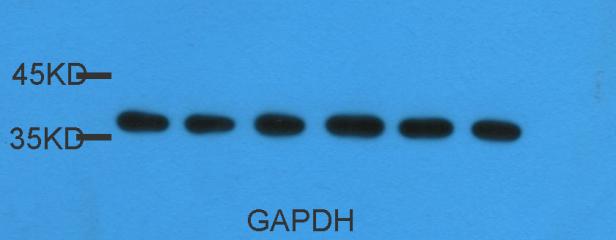


A549


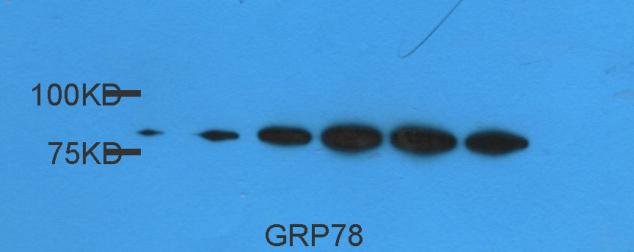


NCI-H520


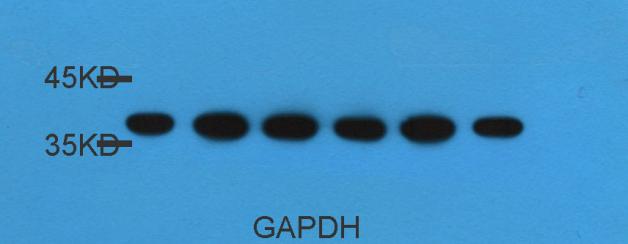


NCI-H520

Figure3B


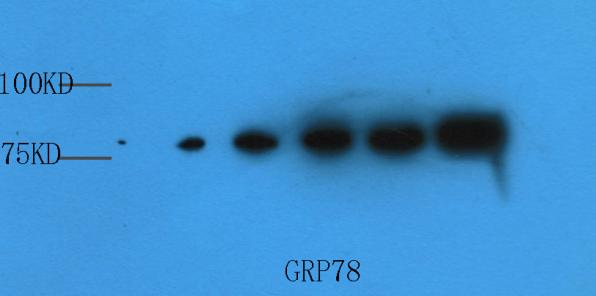


A549


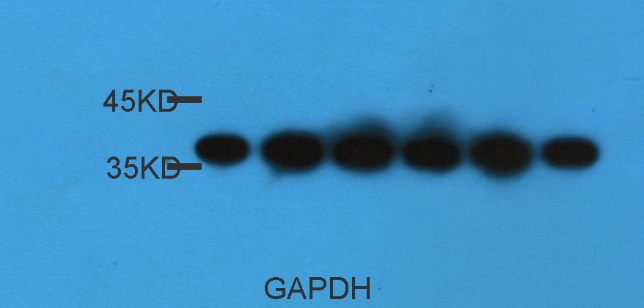


A549


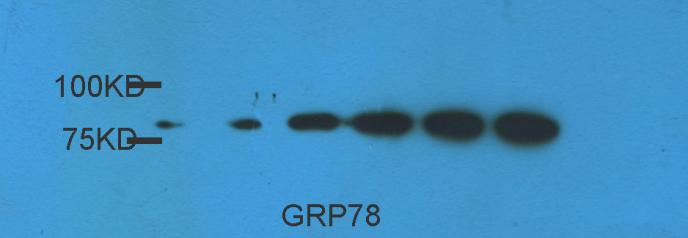


NCI-H520


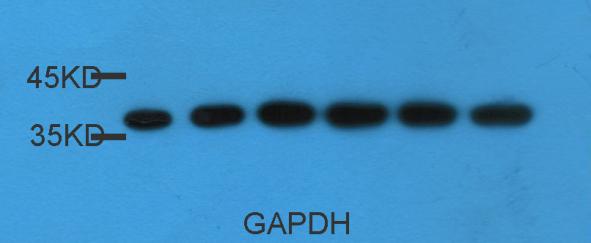


NCI-H520

Figure3E


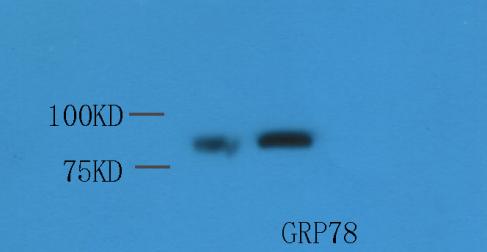

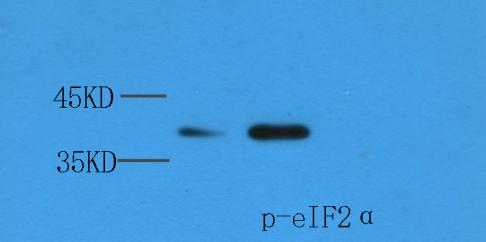


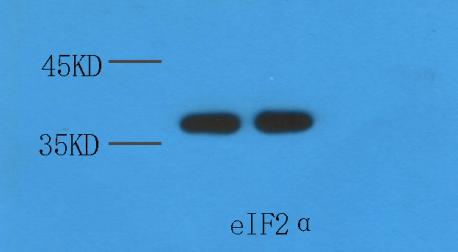

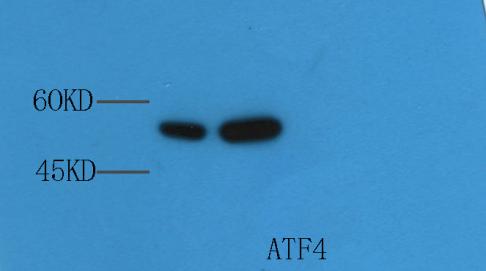


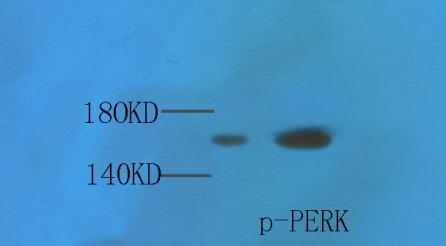

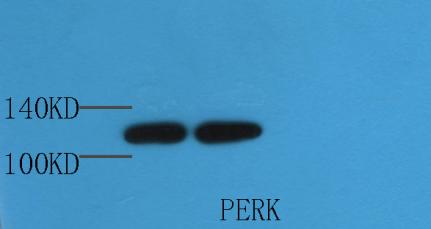


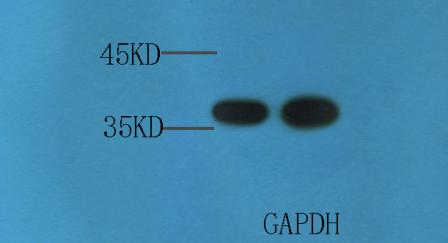


Figure3F


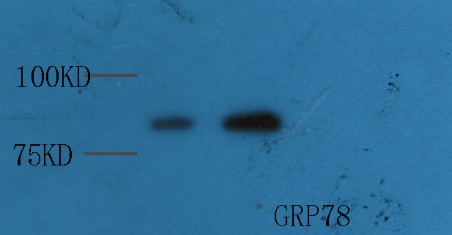

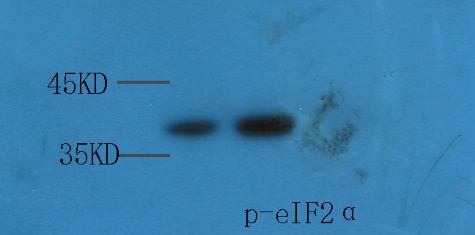


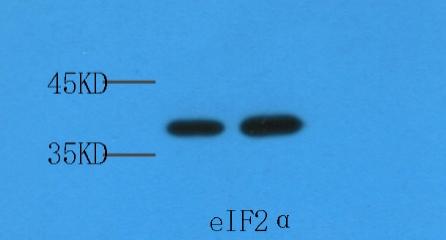

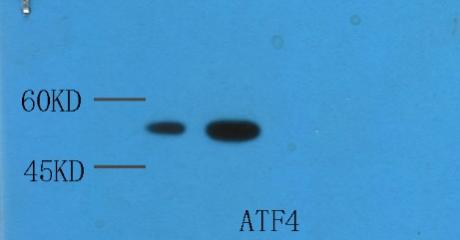


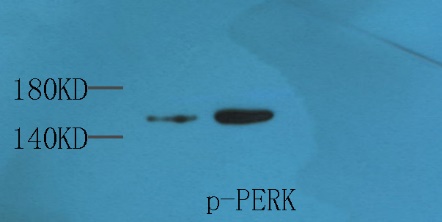

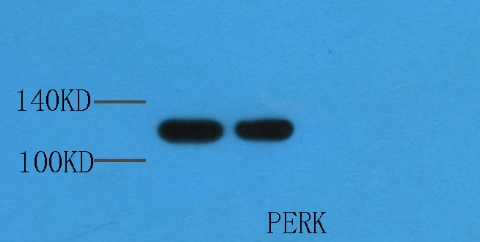


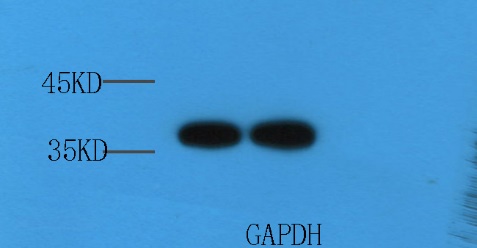


Figure4A


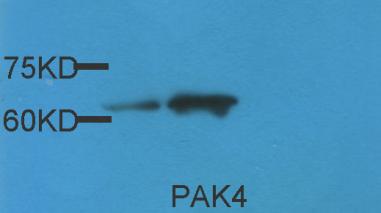

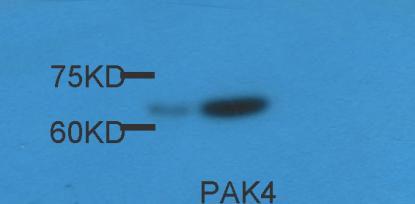
 A549/A549-res NCI-H520/NCI-H520-res


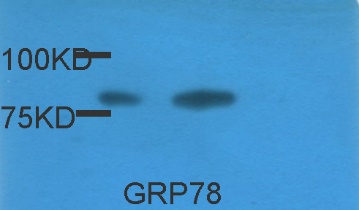

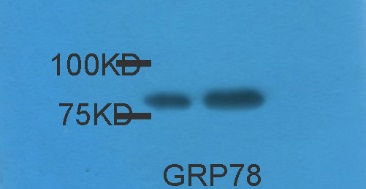


A549/A549-res NCI-H520/NCI-H520-res


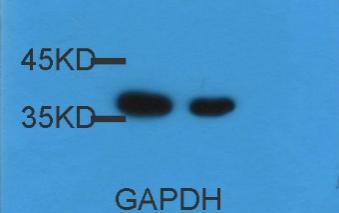

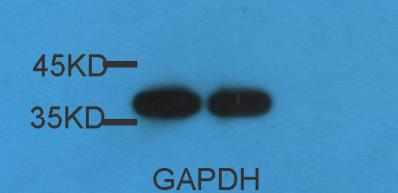


A549/A549-res NCI-H520/NCI-H520-res

Figure4B（left）


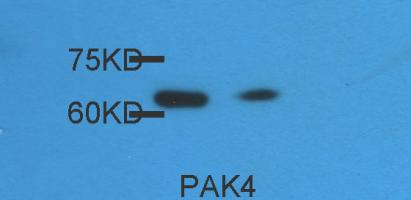

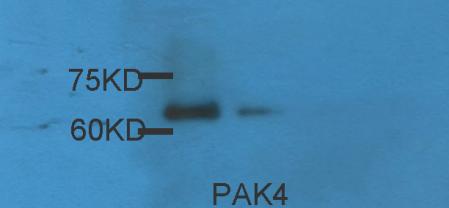


A549-res NCI-H520-res


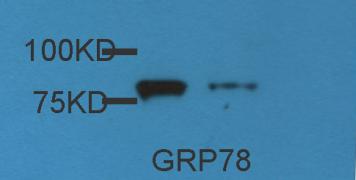

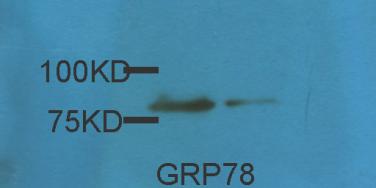


A549-res NCI-H520-res


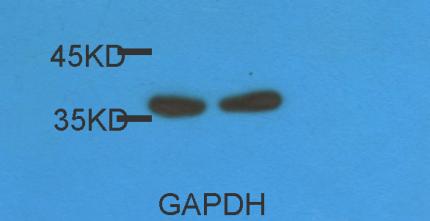

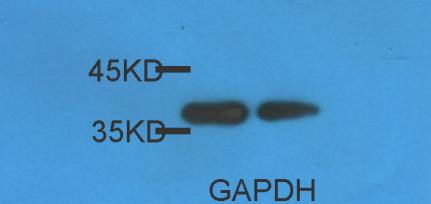


A549-res HCI-H520-res

Figure4B（right）


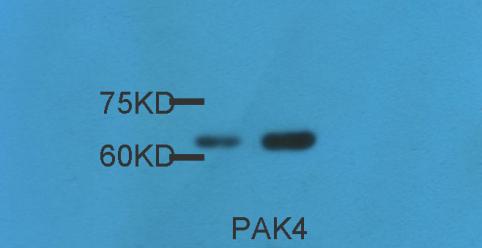

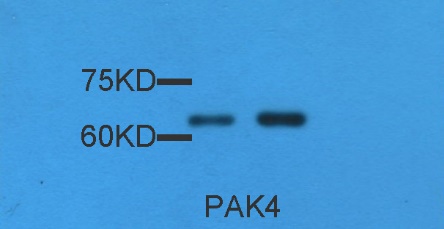


A549 NCI-H520


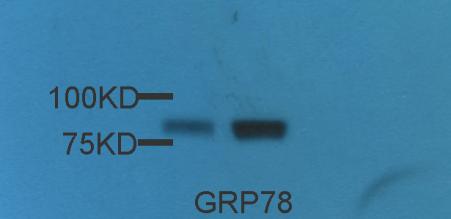

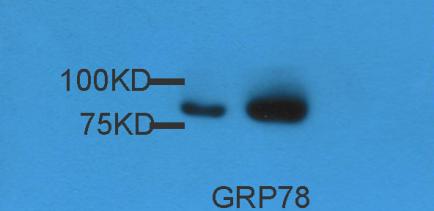


A549 NCI-H520


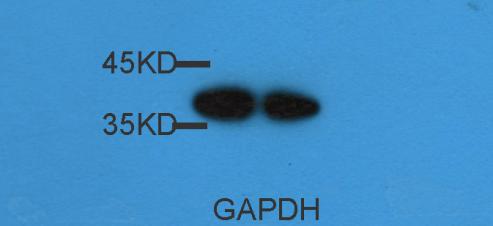

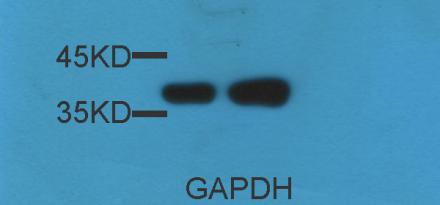


A549 NCI-H520

Figure5D


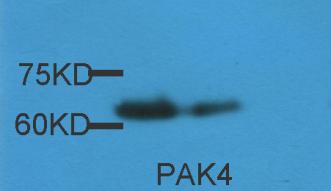

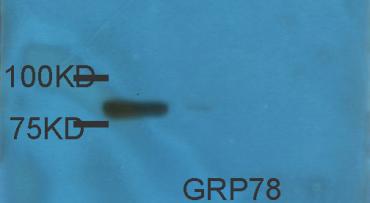

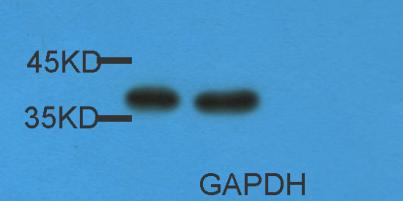


Animal1 Animal1 Animal1


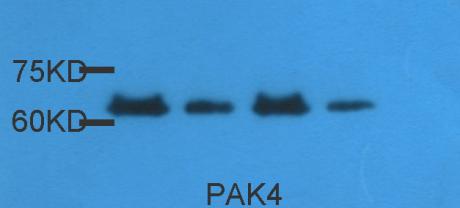

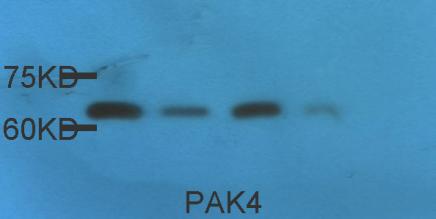


Animal2 Animal3 Animal4 Animal5


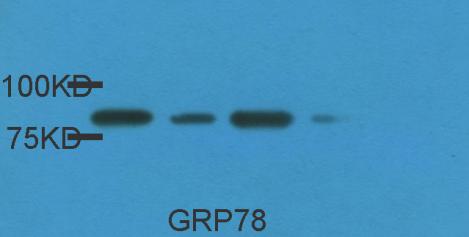

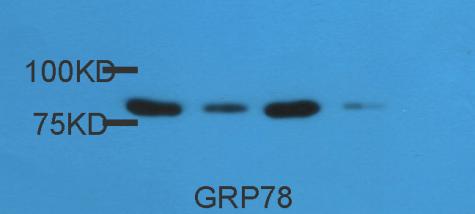


Animal2 Animal3 Animal4 Animal5


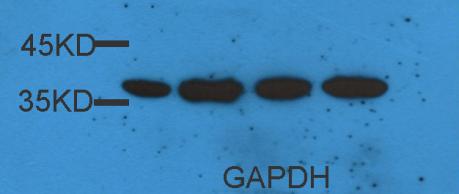

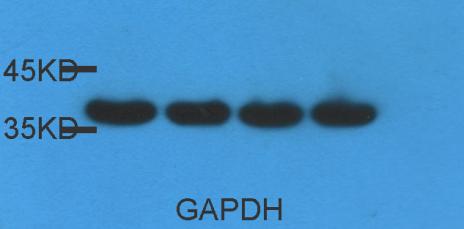


Animal2 Animal3 Animal4 Animal5

Figure7A


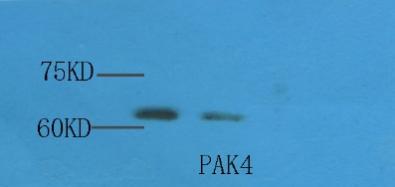

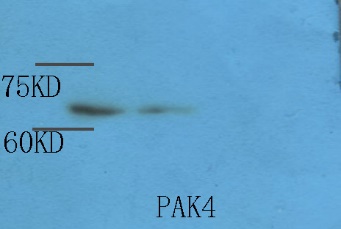


A549-res NCI-H520-res


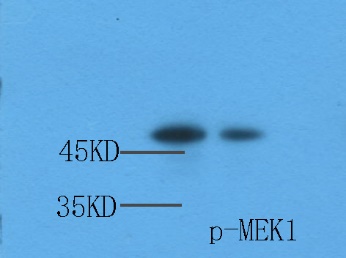

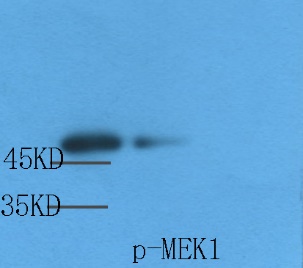


A549-res NCI-H520-res


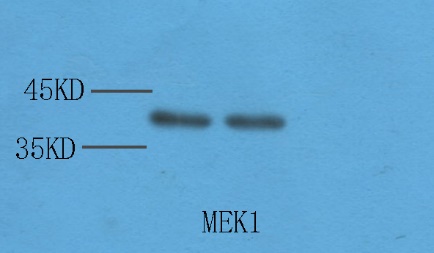

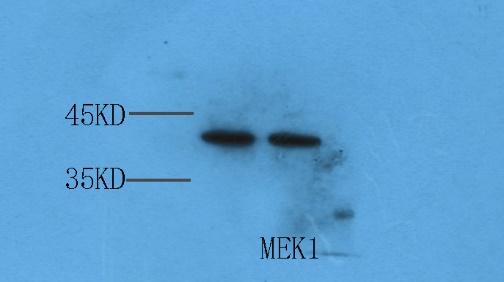


A549-res NCI-H520-res
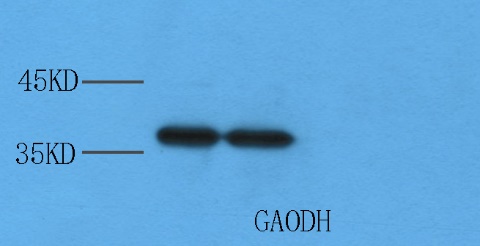

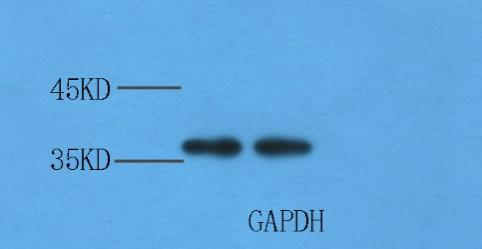


A549-res NCI-H520-res

Figure7D


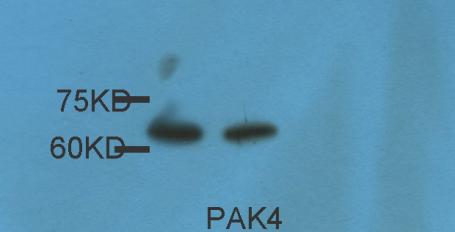

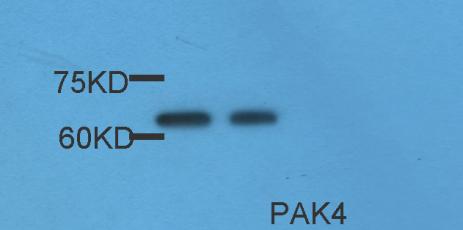


A549-res NCI-H520-res


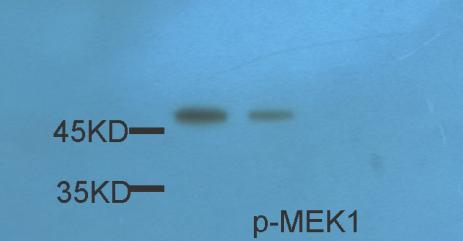

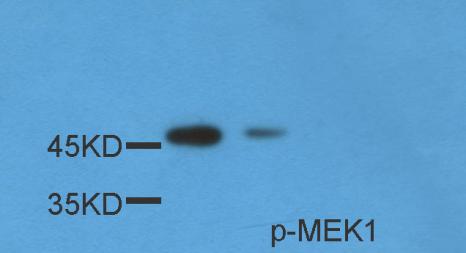


A549-res NCI-H520-res


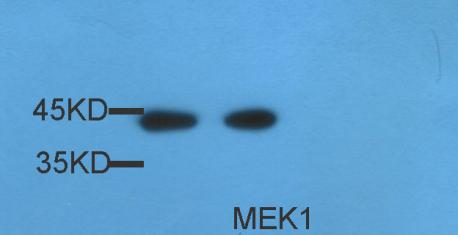

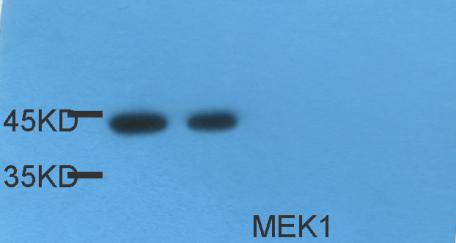


A549-res NCI-H520-res


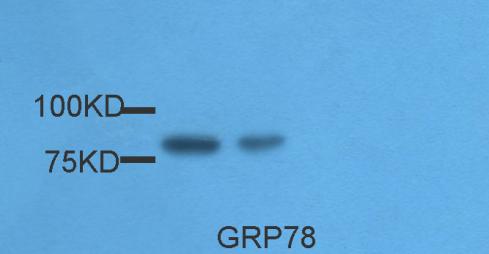

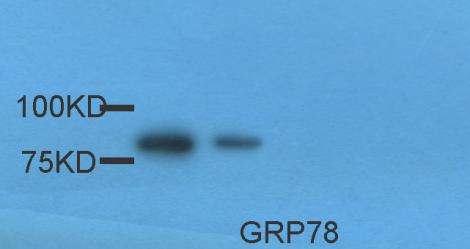


A549-res NCI-H520-res


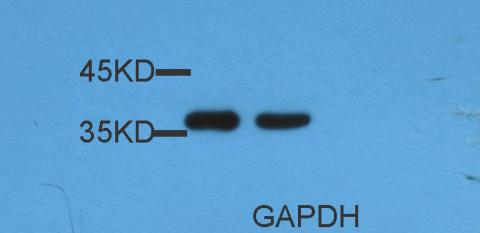

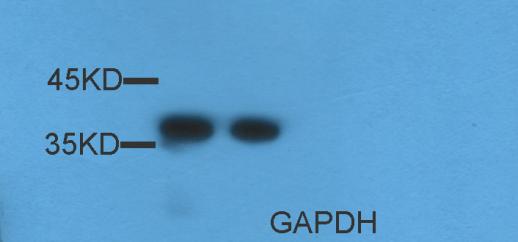


A549-res NCI-H520-res

FigureS1A


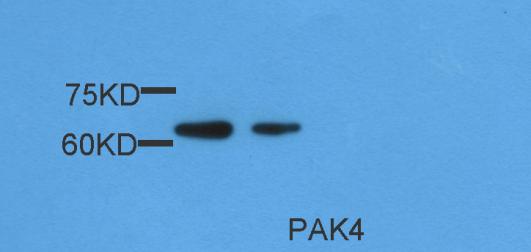

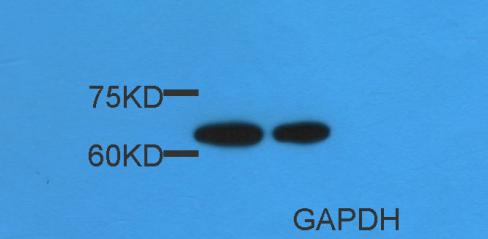


A549-res A549-res


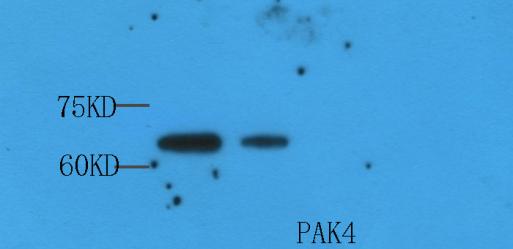

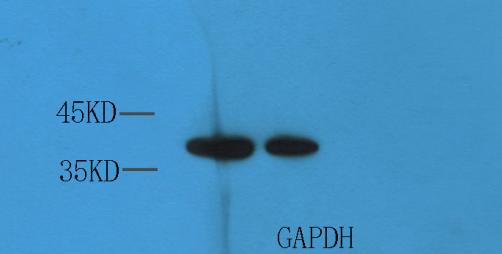


NCI-H520-res NCI-H520-res

Figure7C


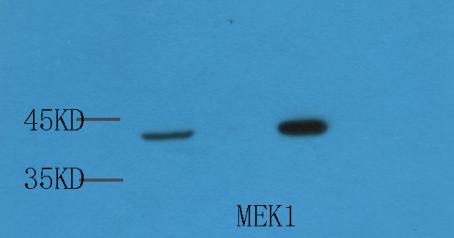

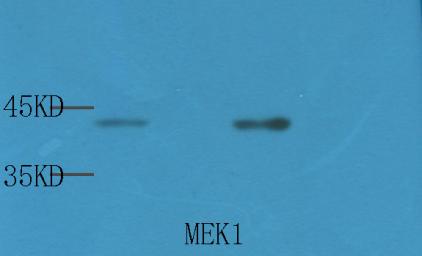


A549-res NCI-H520-res


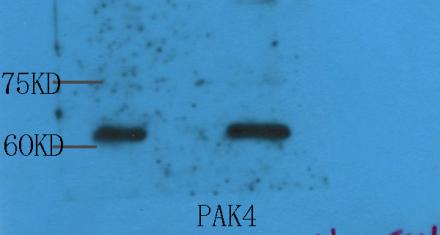

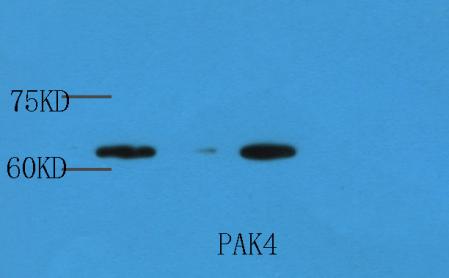


A549-res NCI-H520-res


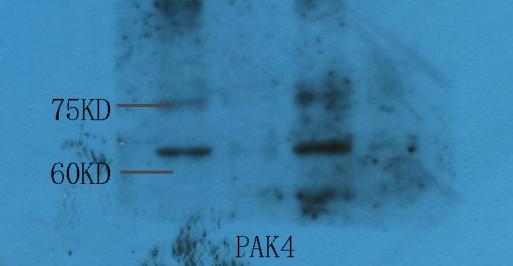

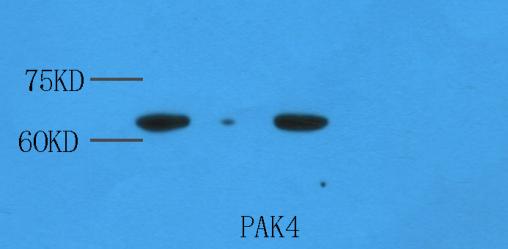


A549-res NCI-H520-res


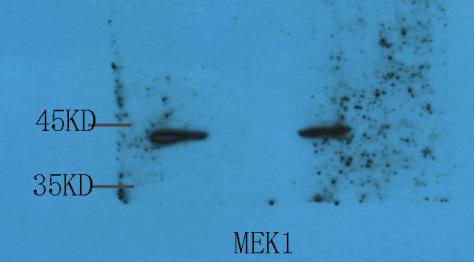

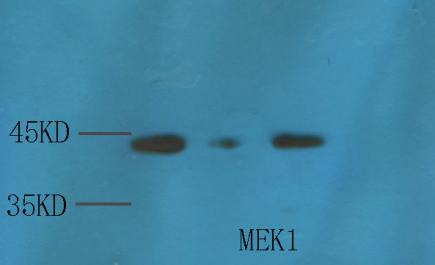


A549-res NCI-H520-res
